# Navigating a stable transition to the age of intelligence: A mental wealth perspective

**DOI:** 10.1016/j.isci.2024.109645

**Published:** 2024-03-29

**Authors:** Jo-An Occhipinti, Ante Prodan, William Hynes, Harris A. Eyre, Alex Schulze, Goran Ujdur, Marcel Tanner

**Affiliations:** 1 Brain and Mind Centre, Faculty of Medicine and Health, University of Sydney, Camperdown, NSW, Australia; 2 Mental Wealth Initiative, University of Sydney, Camperdown, NSW, Australia; 3 Computer Simulation & Advanced Research Technologies (CSART), Sydney, NSW, Australia; 4 School of Computer, Data and Mathematical Sciences, Western Sydney University, Penrith, NSW, Australia; 5 The World Bank, Paris, France; 6 Santa Fe Institute, Santa Fe, NM, USA; 7 Brain Capital Alliance, San Francisco, CA, USA; 8 Baker Institute for Public Policy, Rice University, Houston, TX, USA; 9 Meadows Mental Health Policy Institute, Dallas, TX, USA; 10 Fondation Botnar, Basel, Switzerland; 11 Swiss Academies of Arts and Sciences, Bern, Switzerland; 12 Swiss Tropical and Public Health Institute, Allschwil, Basel, Switzerland

**Keywords:** Public health, Artificial intelligence, Social sciences

## Abstract

In the grand narrative of technological evolution, we are transitioning from the “Age of Information” to the “Age of Intelligence.” Rapid advancements in generative artificial intelligence (AI) are set to reshape society, revolutionize industries, and change the nature of work, challenging our traditional understanding of the dynamics of the economy and its relationship with human productivity and societal prosperity. As we brace for this transformative shift, promising advancements in healthcare, education, productivity, and more, there are concerns of large-scale job loss, mental health repercussions, and risks to social stability and democracy. This paper proposes the concept of Mental Wealth as an action framework that supports nations to proactively position themselves for a smooth transition to the Age of Intelligence while fostering economic and societal prosperity.

## Graphical abstract

**Figure:**
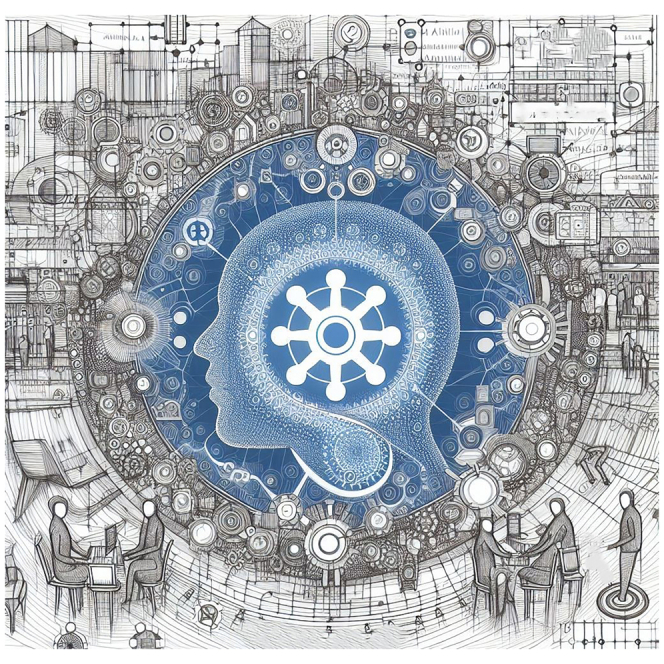

## Disruptive evolution

The pace of advancements in the capabilities of generative artificial intelligence (AI) is unprecedented, raising questions that strike at the heart of societal wellbeing. The Industrial Revolution,^1^ often cited as a historical parallel, unfolded over more than 100 years, allowing societies to adapt and evolve. In contrast, the impending generative AI revolution is happening in a compressed time frame, well within a generation, amplifying the potential for widescale workforce and social disruption. For example, estimates by the Brookings Institution^2^ suggest that in the US alone, approximately 60% of work tasks are facing medium to high exposure to displacement by AI in the coming decades. Other analyses suggest that up to 49% of workers could have half or more of their tasks exposed to replacement by generative AI.^3^ Additionally, there is emerging evidence of diminished earnings among exposed professionals.^4^ Without proactive action from governments and civic organisations, mass unemployment will cast a long shadow over socioeconomic stability, national security, and mental health.

Historically, technological job displacement has brought with it unemployment, but through upskilling and the emergence of new industries and opportunities, new jobs in the economy could be secured. The assumption that the same will also occur in the current wave of AI-driven technological disruption is predominant^5^^,^^6^^,^^7^^,^^8^ but, in our view, unlikely. Unlike previous instances where automation focused on routine tasks, AI’s capabilities extend beyond mere task substitution. Generative AI is an intelligence. It is fast developing the capacity to learn through experience, be creative, adapt, strategize, and generate new knowledge that surpasses human capabilities and inputs.^9^^,^^10^ Additionally, generative AI exists in a broader technological landscape including advancements in computing power, cloud storage solutions, the Internet of Things, and application programming interfaces (APIs) that allow for broader integration into a range of systems, and when combined may amplify socioeconomic impacts.

While timelines are uncertain, domains such as routine data analysis, customer service, accountancy, finance, banking, legal, telemarketing, sales, teaching, commodity trading,^11^ reporting, creative writing, marketing and communications, journalism, and certain medical diagnostic roles such as imagining and the interpretation of lab results (to name a few), are likely to be transformed by AI advancements, where the need for human input will be dramatically reduced.^12^ Additionally, the prospect of generative AI becoming self-sustaining^13^ raises profound questions about the future necessity of human skills in its development. Given this capability, although not all jobs can be replaced, opportunities for participation in the economy are likely to contract considerably. The implications for education systems, traditionally focused on preparing individuals for employment in the economy, are profound and under explored. Education systems will need to adapt^14^ and prepare individuals for a more dynamic and unpredictable environment, where life skills including adaptability, self-directed learning, critical thinking, social responsibility, and civic and digital literacy are essential for success.

Therefore, as we stand on the precipice of a shift in the very nature of work and the focus of education, there is an urgency to reshape our societal frameworks and government policies. The social, political, and economic stability of societies depends on the responsible development and governance of generative AI-based solutions, the extent to which the speed and scale of change can be steered,^15^ and the extent to which new interactions among people, systems, and cultures can emerge.

## Impacts on mental and social wellbeing

In this shifting landscape, public health is a critical concern, in particular, mental health and social wellbeing. The disappearance of familiar job roles and employment insecurity erode the psychological foundations that work provides. Employment not only offers financial security but also fulfills important psychological needs such as time structure, social recognition, a sense of collective purpose, and social connection.^16^ As generative AI threatens to increase unemployment and underemployment, the risk to population mental health is considerable. There is substantial evidence of the link between labor underutilization and suicide rates,^17^^,^^18^^,^^19^ underscoring the profound societal implications of widespread job displacement.

The implications of generative AI’s rise on youth mental health are also profound. In a landscape where AI threatens to reshape job markets at an unprecedented pace, young people face the daunting prospect of preparing for careers that might not exist or might be vastly transformed within the next five years. This may significantly impact young people’s ability to develop a professional profile and social identity, to accumulate capital assets and establish their future economic security. The longer-term impact of diminished economic opportunities for young people was evident following the 2008 global financial crisis. A European Commission analysis found that eight years after the crisis almost one-third of young people remained at risk of poverty or social exclusion.^20^ Similar scarring effects of diminished economic opportunities were reported outside of Europe with young people experiencing a lost decade of income growth, a decline in occupational choices, a decline in employment quality and quantity, and greater exposure to future economic shocks.^21^ The erosion of labor market opportunities for young people could deepen the existing struggle for a sense of purpose and identity that work provides, increasing anxiety, depression, and self-doubt. This raises the question; how do young people prepare themselves for life in the Age of Intelligence? Will AI’s rise render the prevailing trend of narrowly focused vocational specialization obsolete, and will versatility and a broader skill set offer young people better life opportunities?^22^ To remain relevant to societal needs, education systems will need to pivot to deliver broadly applicable social, emotional, innovation, and civic skills, and public-private investments will be needed in social capital infrastructure to provide opportunities for young people to engage in civic participation that nurtures a sense of agency in the face of rapid change.

The challenges posed by generative AI extend beyond individual well-being to the very fabric of society. The concentration of AI capital within a limited number of entities (as frontier firms position themselves to control the market) heightens the risk of job scarcity, declining wages, a contraction in consumption, and business closures, thereby threatening overall economic security and social stability, as rising inequalities creating a landscape ripe for social unrest. Furthermore, the potential for generative AI to concentrate wealth confers unprecedented power to the entities controlling AI technology, thereby weakening democracies. Considering these challenges, two pivotal questions emerge: (i) what role do humans play when substantial portions of the economy are driven by artificial intelligence, and (ii) what new social contract can be forged among businesses, workers, and governments to address these risks. While there is uncertainty regarding the speed of the transition and government responses, what is clear is that the economic status quo is not sustainable, especially in the face of the concurrent threat of climate change. New approaches to these socioeconomic challenges will be essential.

## A new social contract

The social contract is a concept that defines the relationship between individuals and society, in which individuals adhere to rules and norms in return for the benefits of living in an organized and orderly society. With the emergence of generative AI and the potential for significant job displacement, questions arise about how the traditional social contract may need to adapt. Societies will need to consider new arrangements for economic security, education, and the distribution of benefits to ensure that individuals continue to have opportunities for meaningful participation in society. In this context, Mental Wealth^23^ emerges as a critical concept. Mental Wealth is a measure of societal prosperity that quantifies the value of economic *and* social production. It emerged in response to the limitations of traditional measures like GDP in capturing societal prosperity, Mental Wealth expands the boundary of production to include currently unpaid activities that contribute to social prosperity including volunteering, caregiving, environmental protection/restoration, and civic participation.^24^ Social production is the glue that holds societies together, fostering wellbeing and creating socioeconomic, and environmental ecosystems where humans can thrive. In the age of generative AI, social production emerges as a vital avenue for channeling human ingenuity and productive purpose and is a critical but overlooked enabler of the Sustainable Development Goals.

Mental Wealth embraces a comprehensive understanding of prosperity, recognizing that societal well-being is not solely dictated by economic growth, but rather a balance between economic and social production. It represents a departure from conventional economic paradigms and a bold step toward acknowledging the importance of the social fabric in a world increasingly driven by AI-generated productivity. By valuing unpaid forms of labor and incorporating them into economic measurements, we gain a more comprehensive assessment of societal prosperity^25^ that reflects mental health, resilience, and overall cohesion, at regional, national, and global levels. In addition, given generative AI will affect different professions and regions at different scales and time frames, Mental Wealth provides a micro-framework for assessing changes in prosperity.

The destabilizing force of generative AI highlights the urgent need to prioritize the measurement of and investments in social production and social capital infrastructure. As generative AI continues to reshape industries, the potential for job scarcity cannot be ignored. A new social contract is needed. Through the establishment of national Mental Wealth Observatories,^26^ the Mental Wealth Initiative is driving forward the transdisciplinary science needed to support governments, policymakers, and industries to implement innovative solutions to safeguard socioeconomic stability and to make best use of the assets of displaced workforces for societal prosperity. As such, Mental Wealth plays a peace-keeping role, fostering stability, and resilience across systems and cultures.

Among the possibilities, a “social participation wage”^27^^,^^28^ gains prominence in supporting stable transition, offering economic security and an alternative avenue for human skill utilization (including in activities that ameliorate the impacts of climate change), bolstering societal prosperity, and nurturing community resilience. Drawing on the experience of social participation policies in Europe and Japan to support older adults to remain productive,^29^^,^^30^ young people, could be empowered through public-private partnerships that support social production and intergenerational exchange, to become the “city builders”^31^ of our new Age of Intelligence. Such initiatives would not only provide a lifeline to those affected by AI-driven job displacement but also foster a more inclusive, cohesive, and stable society which is the foundation for a thriving economy. A social participation wage for contributions to social production should not be considered charity to those without a paid employment, but rather an alternative primary occupation of equal significance to activities that contribute to economic production.

Humans are inherently social beings,^32^ thriving on mutual dependencies and interdependencies, akin to the biological notion of symbiosis (particularly mutualism where both species benefit). Fostering such symbiotic relationships and guarding against predator-prey dynamics in our economic system becomes paramount for our sustainable coexistence and social stability in this transition to the Age of Intelligence. Just as diverse ecosystems flourish through collaboration and balance, societies too must recognize the interdependencies between the wellbeing of our economic system and that of our social, environmental, and political systems. Despite AI’s disruptive potential, what remains uniquely human is our ability to engage in social production—activities that bind us as a community and strengthen our sense of collective purpose. As the economic landscape shifts at an unprecedented speed globally, capitalizing on these human strengths and investing in strengthening the social fabric as well as in opportunities to contribute to social production becomes a strategic imperative to foster social cohesion, adaptability, shared prosperity, and deepen the Mental Wealth of societies.

This paper solely reflects the views, opinions, arguments of its authors and does not necessarily represent the perspectives of the organizations that authors are associate with.
